# “I Am on Top!”: An Interactive Intervention Program to Promote Self-Regulation Processes in the Prevention of the Use of Doping in Sports High Schools

**DOI:** 10.3390/ejihpe13110183

**Published:** 2023-11-10

**Authors:** Federica Galli, Andrea Chirico, Roberto Codella, Thomas Zandonai, Vindice Deplano, Alessandra De Maria, Tommaso Palombi, Daniel Gotti, Fabio Alivernini, Luca Mallia, Arnaldo Zelli, Fabio Lucidi

**Affiliations:** 1 Department of Movement, Human, and Health Sciences, University of Rome “Foro Italico”, 00135 Rome, Italy; alessandra.demaria@uniroma4.it (A.D.M.); luca.mallia@uniroma4.it (L.M.); arnaldo.zelli@uniroma4.it (A.Z.); 2 Department of Social and Developmental Psychology, “Sapienza” University of Rome, 00185 Rome, Italy; andrea.chirico@uniroma1.it (A.C.); thomas.zandonai@gmail.com (T.Z.); v.deplano@gmail.com (V.D.); tommaso.palombi@uniroma1.it (T.P.); fabio.alivernini@uniroma1.it (F.A.); fabio.lucidi@uniroma1.it (F.L.); 3 Department of Biomedical Sciences for Health, Università degli Studi di Milano, 20133 Milan, Italy; roberto.codella@unimi.it (R.C.); daniel.gotti@unimi.it (D.G.)

**Keywords:** Socio-Cognitive Theory, doping intention, self-regulatory efficacy, moral disengagement, doping knowledge, students

## Abstract

The use of substances to improve sports performance is a widespread phenomenon among adolescents. Several anti-doping programs have been developed, mainly based on knowledge-based evidence. The main aim of the present study was to implement an anti-doping intervention in sports high school students, based on a psychological framework, such as Socio-Cognitive Theory, through the development of a Serious Game (SG), i.e., digital learning based on the game. The experimental design included an intervention group (n = 167; F = 37.7%; Mean_age_ = 17.5 years; SD = 0.58) and a control group (n = 112; F = 42%; Mean_age_ = 17.6; SD = 1). Both of the groups completed the same questionnaire on two different occasions (i.e., time 1 and time 2) for measuring doping intention, self-regulatory efficacy to resist social pressure for the use of substances, moral disengagement, and doping knowledge. Data were analyzed through repeated measures of Group X Time ANOVA, demonstrating some degree of efficacy of the intervention, in particular in terms of the decrease in doping intention and the strengthening of doping knowledge. Moreover, the study demonstrated that the score obtained during the implementation of the SG could partially represent a coherent measure of the participants’ beliefs regarding doping. These results could be considered a starting point for future research to better develop technological anti-doping interventions.

## 1. Introduction

How do we prevent the use of performance- and appearance-enhancing substances? This question has long accompanied anti-doping research, but before analyzing this important issue, it is necessary to define doping. The scientific literature considers doping as a pervasive phenomenon in sport and recreational activities, referring to the use of illegal performance-enhancing drugs and methods to improve performance [1], and the occurrence of one or more of the anti-doping rule violations set (e.g., possession of a prohibited substance or a prohibited method by an athlete or athlete support person [2]). Starting from this definition, the World Antidoping Agency (WADA), its correspondent national organizations, and the international Ministries of Health try to provide an accurate and reliable answer. In this sense, an important reflection regards the switch of the paradigm that guides the anti-doping intervention: from a doping prevention paradigm to a promotion of clean sport behavior [3]. In order to promote the first perspective, widely developed information-based interventions relying on health education and knowledge regarding doping, such as Athletes Training and Learning to Avoid Steroids (ATLAS [4]) and Athletes Targeting Healthy Exercise and Nutrition Alternatives (ATHENA [5]), have been developed. Despite the small impact of these programs in reducing the use of prohibited substances and the declared limited effect of education interventions based on knowledge [6], these types of interventions are still implemented today (e.g., ALPHA program [7]). Moreover, doping prevention programs are also provided through so-called “psycho-educative approaches”. Over time, these types of interventions have been applied (e.g., [8]), focusing on training critical skills to prevent the persuasion of media messages towards doping. In general, media literacy interventions have had positive effects on media literacy skills, even if a recent meta-analysis reported positive but small effects on attitudes and behavioral intentions [9]. In order to take into account the new theoretical paradigm based on the promotion of clean sport, and considering the new strategies and technologies, the present research proposed a digital intervention developing a Serious Game.

### 1.1. Serious Game and Anti-Doping Research

The term “Serious Game” (SG), also known as “digital game-based learning” [10], refers to playful and interactive applications with educational objectives that can foster learning, help acquire new skills, and change behavior [10,11]. In general, gamification refers to the use of game design elements in different contexts [12], and, indeed, SG has been widely used in health areas (e.g., psychology, oncology, education [10]). The use of this type of technology permits us to simulate an experience in terms of environment or system, allowing users to challenge themselves in a less risky or less costly scenario. Another strength of using SG refers to the possibility to try or play again to enhance learning-by-doing and experiential learning [13]. 

Part of the scientific literature on anti-doping research [11,14] has taken into account the innovation in using SG as a learning opportunity that facilitates and stimulates independent and active learning, and improves levels of information and the likelihood of persuasion and attitude and behavior changes [14]. Starting from these considerations, the authors implemented SG programs, such as GAME Project or TARGET, with the aim to transform anti-doping education interventions. Despite the innovative perspective and the positive opinions in terms of usability, enjoyment, and satisfaction [11], these kinds of interventions do not evaluate the psychological variables that underpin doping use. Indeed, an important meta-analysis by Ntoumanis and colleagues strongly recommended developing and implementing doping-related interventions based on psychological frameworks [6].

### 1.2. The Present Study

In light of the above and considering the gaps in the literature, the present study implemented an SG intervention to promote clean sport behavior in sport high school students, considered a risk population in relation to using substances to improve sport performance and/or to enhance appearance (e.g., [15,16]). Following Ntoumanis’ suggestions [6], this study considered Socio-Cognitive Theory variables [17] in examining the SG outcomes. In particular, the present research evaluated how an innovative and technological anti-doping program could influence the critical social–cognitive factors in referring to the decision to use substances, such as intention to use substances. More precisely, the intervention took into consideration self-regulatory efficacy in resisting social pressure towards the use of substances, and moral disengagement. This latter refers to the cognitive process that individuals can apply to deactivate moral self-regulation and disengage from moral norms, while apparently avoiding guilt and/or self-censure, through personal maneuvers by which moral self-sanctions can be disengaged. Specifically, the moral justification (i.e., a person’s detrimental conduct may be deemed acceptable because it serves socially valid or moral purposes); exonerative comparison (i.e., comparison with more flagrant inhumanities); and displacement and diffusion of responsibility in order to remove personal responsibility were taken into account [18]. Moreover, we also evaluated the knowledge regarding the anti-doping system, the assumption of substances and/or supplements, and the rules in sport contexts. 

A further aim of the present research was to evaluate how and if the scores obtained at the end of the SG could represents the individuals’ internal beliefs, coherently with the answers and the opinions about the doping issues, as evaluated using the questionnaires.

## 2. Materials and Methods

### 2.1. Design

The principal design applied in the present research was a quasi-experimental longitudinal design [19]. Indeed, the target dependent variables were measured before and after the intervention [20]. The pretest–posttest design consisted of two groups: the “intervention group” and the “control group”. The first group received the intervention and the second did not [20].

### 2.2. Participants

Six Italian sports high schools were informed about the aims of the present research and were recruited. The recruitment process was undertaken to construct a convenience sample considering the locations of the schools, which were close to the projects’ partner (i.e., three schools from Rome and three from Milan), and the inclusion criterion was being a “sports” high school. The study was approved by the Ethics Review Board of the Department of Social and Developmental Psychology, “Sapienza” University of Rome (n. 0001020-28/07/2022). Sport high school students participated in the study after having given written consent (if minors, parents provided consent), and they were recruited using a convenience sampling procedure for the selection of two distinct groups: “intervention group” and “control group” (following the convenience assignment). The “intervention group” was composed of 167 students (37.7% female) who actively participated in SG activities, and the “control group” was composed of 112 students (42% female) who only contributed to the questionnaire assessment. The groups’ characteristics are reported in Table 1, showing that there is no significant difference between the “intervention group” and “control group” in terms of age, gender or sport type, but there was for sport level.

### 2.3. Procedure, Intervention and Its Implementation

The intervention was developed and coordinated by the University of Rome “Sapienza”, the University of Rome “Foro Italico”, and the University of Milan “La Statale”. The content of the SG was co-designed by psychologists with expertise in sports doping, sport scientists, and a computer scientist. The sport psychologists provided expertise in understanding the psychological aspects and defining specific objectives for the game. The sport scientists integrated scientific principles and best practices into the SG design, bringing in their knowledge of exercise physiology, nutrition, and other relevant fields to ensure that the in-game actions and scenarios are aligned with the latest research and recommendations, and are close to real-life sports scenarios. The computer scientists, including game developers and designers, worked on the technical aspects of the SG. They created the interactive environment, designed the user interface, and programmed the game mechanics. The collaboration with the sports psychologists and sport scientists ensured that the game elements aligned with the target psychological variables. With the aim of creating a realistic storyboard related to a credible situation in sport, a focus group composed of the target population was organized. Focus groups consisting of athletes and high school students provided feedback on initial prototypes of the SG, evaluating the game’s usability, relevance, and effectiveness. This feedback was gathered through structured discussions. 

The SG reported the everyday life over four weeks of a track and field athlete who experiences different situations leading up to running in an important competition. The main character interacts with his girlfriend, parents, coach, and teammate with whom he faces different decisions and challenges related to the possibility of using doping substances or supplements. The protagonist has the option to make different decisions and, based on them, the climax of the story presents different scenarios (e.g., the athlete decides to not use doping and wins the race; the athlete denounces the teammate; the athlete consumes banned substances, etc.). Once the research group finished writing the storyboard, the computer scientist developed the SG with the support of the graphic designer and voice actors, under the weekly supervision of all the units. 

The intervention comprised four 90 min sessions, spread over about a month during school lessons and relying on a trained sport psychologist, divided as follows:

1. First Session (Introduction and Pre-Assessment)—In the first 90 min session, the sport psychologist introduced the project to the participants. This likely included an overview of the goals and objectives of the intervention. The sport psychologist initiated the session by explaining the purpose of the intervention and the importance of the participants’ involvement. Participants were asked to answer a questionnaire at the beginning of the session (time 1), which could be used to give a baseline assessment of their knowledge, attitudes, or behaviors related to the intervention’s topic.
2. Second Session (Definition and Platform Introduction)—During this session, the sport psychologist provided a comprehensive definition of SG and explained how to use the platform as part of the intervention. The sport psychologist likely engaged the participants in discussions and activities related to the definition and usage of the platform. They may have facilitated discussions, answered questions, and ensured that participants had a clear understanding of the topic and the tools they would be using.
3. Third Session (Game Score Discussion)—In the third session, the sport psychologist delved into the participants’ scores obtained at the end of the SG. This may have included discussing the significance of these scores, interpreting them in the context of the intervention, and exploring potential implications. The sport psychologist would have initiated and facilitated a discussion about the participants’ scores. This would likely have been an interactive session wherein participants could share their experiences, ask questions, and express their thoughts and concerns related to their scores. The psychologist’s role would have been to guide these discussions and ensure that participants were actively engaged.
4. Fourth Session (Anti-Doping Rules and Post-Assessment)—In the fourth and final session, the sport psychologist explained the anti-doping rules and the WADA code, which are relevant to the context of the intervention. This session may have also included a discussion of the importance of these rules. The sport psychologist answered any remaining questions participants had about the intervention, the topic, or the anti-doping rules. Similar to the first session, participants answered a questionnaire at the end of this session (time 2), which could be used for post-assessment and to measure any changes in knowledge, attitudes, or behaviors following the intervention.

### 2.4. Instruments

Both groups provided data by answering the same questionnaire on two occasions, separated by around a month. The “intervention group” filled out the questionnaire before and after the intervention sessions and, in addition, obtained SG scores for each Socio-Cognitive Theory variable. The questionnaires used have been selected since they were the most used questionnaires for measuring socio-cognitive constructs in the scientific doping literature [21].

#### 2.4.1. Doping Intention

In line with previous studies (e.g., [22]), the doping intention was measured by asking students to report their doping likelihood in the context of two hypothetical scenarios, within which they had to decide whether or not to use illegal substances. The supposed situations featured two of the most common reasons for consuming banned substances: enhancing performance the day before the most important competition of the season, and aiding recovery from an injury that compromises participation in the most important competition of the season. In both cases, participants reported their likelihood of using doping by ranking three items for each scenario on a 7-point Likert scale from 1 (“Not at all likely”) to 7 (“Very likely”). The internal consistency reliabilities were 0.92 and 0.90 for time 1 and 0.94 for time 2 in both the “intervention group” and “control group”, respectively.

#### 2.4.2. Self-Regulatory Efficacy to Resist Social Pressure for the Use of Substances

Participants ranked seven items, measuring whether or not they felt confident in avoiding social pressure towards the use of banned substances. Each item was introduced by the same stem (“How capable do you feel of avoiding the use of prohibited substances…) and was presented on a 7-point scale ranging from 1 (“Not at all confident”) to 7 (“Completely confident”). The internal consistency reliabilities were 0.93 and 0.95 for time 1 and 0.96 for time 2 in both the “intervention group” and the “control group”, respectively.

#### 2.4.3. Moral Disengagement

The measurement of doping moral disengagement relied on six moral disengagement mechanisms, selected following previous studies (e.g., [15]). Students answered by reporting, for situations in which the use of doping substances was suggested, their agreement as to whether it should or should not be condemned (e.g., “Those who use illicit substances in sport are not to be blamed, but those who expect too much from them should”). For each item, participants rated their agreement on a 5-point scale ranging from 1 (“I do not agree at all”) to 5 (“I completely agree”). The internal consistency reliabilities were 0.39 and 0.75 for time 1 and 0.75 and 0.78 for time 2 in the “intervention group” and “control group”, respectively.

#### 2.4.4. Doping Knowledge

Knowledge was evaluated by selecting 10 items from the 40 on the “WADA’s Play True Quiz”. This test measures knowledge in terms of the anti-doping system, choosing for each statement “True” or “False”, with a total score range of 0 to 10.

### 2.5. Data Analysis

Descriptive characteristics (i.e., age, gender, type, and level of sport) were examined using statistical analysis, means with standard deviations, and percentages, and were analyzed by analysis of variance (ANOVA) and chi-square tests as relevant. The reliability (i.e., Cronbach’s alpha) of the target measures was examined. Then, a series of repeated measures ANOVA tests were performed for each variable to evaluate differences across time (“time 1” vs. “time 2”) and between study groups (“intervention group” vs. “control group”), and for the interaction between time and study group. Furthermore, model 4 of the PROCESS macro [23] was selected for mediation analyses, assessing SG score as the mediator in the relations between “time 1” and “time 2” variables scores (i.e., intention, self-efficacy, and moral disengagement). The aim of the mediation analyses was to explore and quantify the intermediate processes that explain the relationship between psychological constructs evaluated by questionnaires and the choices made during the SG. The *p*-value was established as <0.05. Confidence intervals (95%) were estimated with 10,000 bootstrap samples and unstandardized regression coefficients. The effects were considered significant if their confidence intervals did not include zero. All the analyses were conducted using the SPSS software (version 28.0).

## 3. Results

### 3.1. Reliability of the Measures

In terms of the reliability, the alpha coefficients of all the measurements support the internal consistency of the item sets for both times of the research: time 1—from 0.75 to 0.95; time 2—from 0.75 to 0.96. The only exception is associated with the moral disengagement variable at time 1 in the intervention group.

### 3.2. Doping Intention

**Figure 1:**
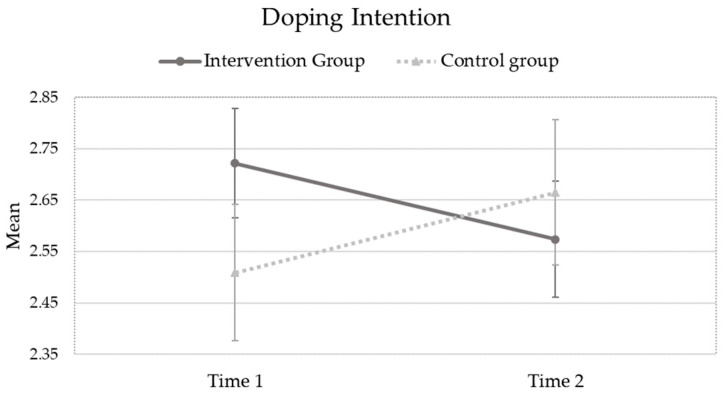
Students’ doping intention across experimental conditions and across timepoints.

The results of a first repeated-measures ANOVA considering students’ likelihood to use doping as a dependent variable have revealed a statistically significant “Group by Time” effect (F_(1,272)_ = 3.89; *p* = 0.05; partial eta square = 0.014). As can be seen in Figure 1, students’ intention to dope decreased over time for the “intervention group” (mean (time 1) = 2.72; SD (time1) = 1.39; and mean (time 2) = 2.57; SD (time2) = 1.44), and increased over time for the “control group” (mean (time 1) = 2.51; SD (time1) = 1.37; and mean (time 2) = 2.67; SD (time2) = 1.47). However, there were no statistically significant main effects of “Time” (F_(1,272)_ = 0.003; *p* = 0.958; partial eta square < 0.001) and “Group” (F_(1,272)_ = 0.147; *p* = 0.702; partial eta square = 0.802).

### 3.3. Self-Regulatory Efficacy in Resisting Social Pressure towards the Use of Substances

A second repeated-measures ANOVA, which considered students’ self-efficacy as the dependent variable, yielded no statistically significant effect for the “Group by Time” interaction (F_(1,272)_ = 0.004; *p* = 0.952; partial eta square < 0.001) and the main effect of “Group” (F_(1,272)_ = 1.632; *p* = 0.202; partial eta square = 0.006). However, there was a significant main effect of “Time” (F_(1,272)_ = 10.549; *p* < 0.01; partial eta square < 0.037). When these effects were more carefully examined via pairwise comparisons, differences emerged across timepoints. The mean levels of self-efficacy significantly decreased over time in both “intervention group” students (F_(1,272)_ = 7.006, *p* = 0.009; partial eta square = 0.025; mean (time 1) = 5.52; SD (time1) = 1.54, mean (time 2) = 5.12; SD (time2) = 1.86) and “control group” students (F_(1,272)_ = 4.168, *p* = 0.042; partial eta square = 0.015; mean (time 1) = 5.28; SD (time1) = 1.82, mean (time 2) = 4.89; SD (time2) = 1.88).

### 3.4. Moral Disengagement

Similarly, a third repeated-measures ANOVA, which considered students’ moral disengagement as a dependent variable, showed no statistically significant effect for the “Group by Time” interaction (F_(1,277)_ = 1.349; *p* = 0.246; partial eta square = 0.005) and the main effect of “Group” (F_(1,277)_ = 1.705; *p* = 0.193; partial eta square = 0.006). However, there was a significant main effect of “Time” (F_(1,277)_ = 11.570; *p* < 0.01; partial eta square = 0.04). A detailed examination of the pairwise comparisons revealed a significant effect among “intervention group” students (F_(1,277)_ = 12.697; *p* < 0.001; partial eta square = 0.045), whereas this pattern did not emerge for their “control group” counterparts (F_(1,277)_ = 2.095; *p* = 0.149; partial eta square = 0.008). Over time, “intervention group” students showed a reduction in their average level of doping moral disengagement (mean (time 1) = 1.77; SD (time1) = 0.51; and mean (time 2) = 1.61; SD (time2) = 0.62), whereas “control group” students’ moral disengagement decreased to a lesser extent (mean (time 1) = 1.64; SD (time1) = 0.64; and mean (time 2) = 1.57; SD (time2) = 0.62).

### 3.5. Doping Knowledge

**Figure 2:**
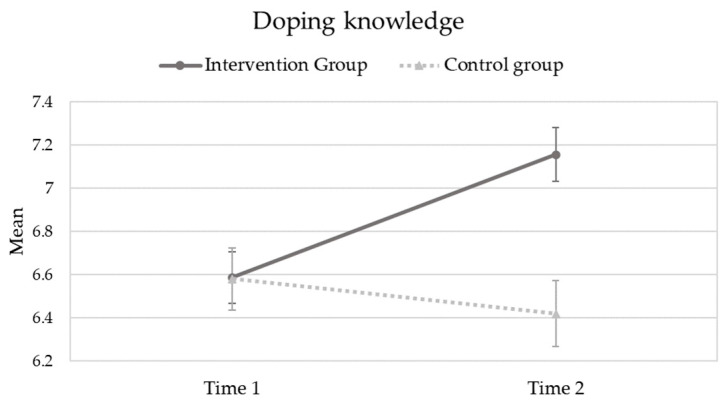
Students’ doping knowledge across experimental conditions and across timepoints.

Finally, the results of the last repeated-measures ANOVA considering students’ doping knowledge as the dependent variable revealed a statistically significant “Group by Time” effect (F_(1,277)_ = 10.211; *p* = 0.002; partial eta square = 0.036) and the main effect of “Group” (F _(1,277)_ = 5.758; *p* = 0.017; partial eta square = 0.020). To visualize the “Group by Time” effect, Figure 2 shows the students’ knowledge mean scores across experimental conditions and across timepoints. Consistent with the figure, students’ doping knowledge increased over time for the “intervention group” (mean (time 1) = 6.59; SD (time 1) = 1.37; and mean (time 2) = 7.16; SD (time 2) = 1.38) and decreased over time for the “control group” (mean (time 1) = 6.58; SD (time 1) = 1.74; and mean (time 2) = 6.42; SD (time 2) = 1.92). Nevertheless, there was no statistically significant main effect of “Time” (F_(1,272)_ = 3.196; *p* = 0.075; partial eta square < 0.011).

### 3.6. Mediation Analyses

**Figure 3:**
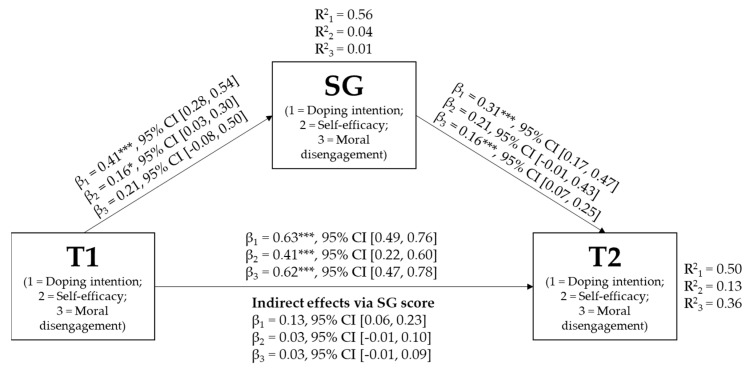
Coefficients of the three mediation models tested in the intervention group. Note. * *p* < 0.05, *** *p* < 0.001; CI = confidence interval; T1 = all variables’ scores collected at time 1; SG = all scores obtained in the Serious Game; T2 = all variables’ scores collected at time 2.

To test whether changes in “intervention group” students’ intentions in relation to doping, self-efficacy, and moral disengagement from time 1 (i.e., before intervention) to time 2 (i.e., after intervention) were mediated by SG scores collected during the intervention (i.e., second session), mediation models were tested separately for each variable. Figure 3 summarizes the three models’ statistical information. Regarding the direct effects, all variables’ scores collected at time 1 significantly and positively predicted their scores collected at time 2. All time 1 variables’ scores, except for the time 1 moral disengagement score (β = 0.21, 95% CI: (−0.08, 0.50)), significantly and positively predicted their SG scores. Lastly, all SG scores, except for SG self-efficacy score (β = 0.21, 95% CI: (−0.01, 0.43)), significantly and positively predicted their time 2 scores. Regarding the indirect effects, only time 1 doping intention score had a significant indirect positive effect on time 2 doping intention score via SG doping intention score (β = 0.13, 95% CI: (0.06, 0.23)). Thus, the time 1 doping intention score positively predicts the SG score, which, in turn, positively predicts the time 2 score.

## 4. Discussion

The present study aimed to answer the call of WADA regarding the issues most related to susceptibility to consuming banned substances on the part of young athletes, who might consider doping as a shortcut to achieving sport goals [24]. The main intent was to develop an anti-doping intervention that could take into account scientific suggestions, such as overcoming the classical educational programs in favor of psychological ones [25], focusing on theoretical frameworks [6] and evaluating the effects of technological anti-doping interventions [14]. On this basis, the findings suggest that this SG-based anti-doping intervention was able to influence doping intention, moral disengagement, and doping knowledge in sport high school students. The SG intervention, however, did not influence the effectiveness of self-regulatory efficacy to resist social pressure towards the use of substances. Finally, the SG players’ choices truly represented the students’ beliefs regarding doping. These findings are discussed below, as they suggest certain conclusions.

As a first aim, the study evaluated the effectiveness of the implemented intervention by assessing the impacts of Socio-Cognitive Theory constructs (i.e., doping intention, self-regulatory efficacy to resist social pressure for the use of substances, and moral disengagement) and doping knowledge, testing the questionnaire’s answers at time 1 and at time 2 for both intervention and control groups. The results show that the proposed intervention tended to reduce the likelihood of dope-use in students who took part in the intervention. This aspect is particularly important when considering that doping intention [26], temptation [27] or likelihood [22] are proxies for doping behavior. By demonstrating a reduction in these proxies, this intervention suggests a potential positive impact on the students’ real-world decisions related to doping. 

Furthermore, it is interesting to note that pairwise comparisons highlighted relevant changes in the moral disengagement construct. In this sense, the intervention seemed to have an effect in terms of empowering sport high school students’ choices and revealing the consequences when they decide to use, or not, banned substances. Also, in this case, this result was particularly important, as most of the scientific literature demonstrated how adolescents who declared high levels of doping moral disengagement typically tended towards doping [15]. Indeed, more recently, scholars have underlined the relevance of focusing on moral interventions in order to promote “clean sport behavior” (e.g., to not use doping [28]). However, the proposed intervention was not sufficient to have a significant impact on increasing self-regulatory efficacy as regards resisting social pressure towards the use of substances, as shown in similar research studies undertaken in different contexts [29]. In order to obtain a change in a psychological construct that strongly depends on social aspects, it could be necessary to emphasize social skills, and to consider dealing with assertiveness and/or self-esteem. 

As previously declared, the present study evaluated not only the Socio-Cognitive Theory variables, but also the possible increase in doping knowledge. Students in the intervention group already possessed different knowledge about doping, as they studied the history of doping, the rules of WADA, and anti-doping as school subjects in class, and most of them were athletes (n = 86.2%). This notwithstanding, the findings show that the intervention allowed them to acquire more developed notions.

In regard to the other aim of the present research, we explored how the participants’ decisions, evaluated through the SG scores, could really represent the beliefs of the students with respect to doping issues, as given by the questionnaire answers. As the results indicate, the hypothesized mediation models show that the SG scores seem to offer a coherent measure expressing the participants’ opinions, especially in terms of doping intention. This alignment suggests that the choices made within the SG provide an accurate representation of the participants’ inclinations toward doping. In other words, the SG effectively captured the essence of their intentions related to doping, offering an additional dimension of insight beyond traditional questionnaire responses, and this is coherent with the results of previous reviews of the literature on the topic [30]. As concerns the non-significant paths related to self-regulatory efficacy and moral disengagement, these results could be explained from both theoretical and statistical points of view. Regarding these two psychological constructs, it could be necessary to extend the measurement during the SG’s questions to better explore these aspects (e.g., creating more dilemmas). Statistically speaking, these paths could have a significant effect in increasing the number of participants. Expanding the number of participants could help us to better detect the complexity of these psychological constructs and their interplay with the decisions made in the SG.

In sum, the present study contributes to an emerging body of research that aims to address the challenges faced by young athletes in resisting the temptation of doping and making ethical decisions in sports contexts. While previous studies have touched upon various aspects of anti-doping interventions [8], this research stands out for several reasons. First of all, the use of SG as an intervention tool represents an innovative approach in the anti-doping field. The SG provided a dynamic and engaging platform for participants to navigate dilemmas related to doping, allowing for a more nuanced exploration of their attitudes and intentions. This methodology sets this research apart from many previous studies that relied solely on traditional educational programs [6]. In addition, the observed changes in moral disengagement highlight the importance of addressing moral dimensions in anti-doping interventions. This aligns with recent scholarly emphasis on the role of moral interventions and moral identity in preventing doping behavior [28]. Finally, the intervention increased doping knowledge among participants, even for those with existing baseline knowledge. This suggests that anti-doping education can be further enriched through innovative approaches. 

### Strengths, Limitations and Future Research 

The present research has several strengths. First, to our knowledge, this study is the first structured SG intervention regarding doping issues implemented amongst sport high school students, a group that is particularly at risk for PAES use [15]. The SG programs already in use (e.g., GAME Project [31]) do not evaluate the effectiveness of these types of technological interventions; in addition, this study estimated for the first time, through the mediation of the SG score, the role and the value of the SG as a measure of the participants’ beliefs. These strengths notwithstanding, there exist some limitations that should be noted. First, the investigation was implemented in high school settings, and, as a result, the two samples of sports high school students were not stratified. Therefore, the findings should not and cannot be generalized to the broader sports high school student population. Second, students’ assignments to the two conditions of the program (i.e., “control group” vs. “intervention group”) were not rigorously randomized, but were based on the schools’ availability using a convenience sampling procedure. Third, there were methodological concerns that need to be mentioned: the number of students involved in the study and the possibility that the findings might be biased, and be a function of the school context. Moreover, a significant limitation of this study is the low reliability index associated with moral disengagement variable at time 1 only for the intervention group. This unreliable attribute may have introduced a measurement error, potentially leading to inaccurate results. This limitation hinders the study’s ability to draw robust and generalizable conclusions; however, the data consistency and reliability indexes overall range from 0.75 to 0.96. This low reliability index could be due to the application of the statistics on a single small subset of participants (only intervention group a time 1).

In conclusion, future studies should extend SG interventions to other sport high school and/or to other sport contexts, developing an SG intervention focused on a specific sport or discipline (e.g., SG sets in a sport for sport-specific athletes) and/or other populations with stakes in the sport and doping issues (e.g., coaches, trainers, parents). Moreover, future research could develop an SG intervention that increases the specific dilemmas that arise in relation to each psychological variable, and try to measure the changes in socio-cognitive variables with time through long-term follows up, as recently suggested by [25].

## 5. Conclusions

The present study provides empirical support for the effectiveness of an anti-doping intervention based on a Serious Game, i.e., a persuasive videogame with the scope to prepare the users to face different situations related to the decisions surrounding and the consequences of using banned substances, in sport high school students. It has made significant strides in the development of anti-doping interventions for young athletes, offering valuable insights and promising results. However, ongoing research is needed to refine and expand upon these findings, addressing the identified limitations and working toward more effective strategies to prevent doping among the next generation of athletes.

## Figures and Tables

**Table 1:** Characteristics of the intervention and control groups.

	Intervention Group	Control Group		
	N = 167 M (SD) %	N = 112 M (SD) %	F χ2	*p*
Age	17.51 (0.58)	17.65 (1)	2.27	0.13
Gender			1.75	0.42
Male	61.1	58		
Female	37.7	42		
N.a.	1.2	/		
Sport type			2.2	0.53
Team	47.3	48.2		
Individual	38.9	33.9		
Physical activity (e.g., gym, fitness, etc.)	9.6	9.8		
No sport	4.2	8.0		
Sport level			18.13	<0.01
Amateur	10.2	15.2		
Local	3.6	13.4		
Regional	37.1	35.7		
National	38.9	19.6		
International	6.0	7.1		
N.a.	4.2	8.9		

Note. M: mean; SD: standard deviation; N.a.: Not avaiable.

## Data Availability

The data presented in this study are available on request from the corresponding author.
